# A silver bullet? The role of radiology information system data mining in defining gunshot injury trends at a South African tertiary-level hospital

**DOI:** 10.4102/sajr.v25i1.2018

**Published:** 2021-03-02

**Authors:** Dale K. Creamer, Asif Bagadia, Clive Daniels, Richard D. Pitcher

**Affiliations:** 1 Division of Radiodiagnosis, Department of Medical Imaging and Clinical Oncology, Faculty of Medicine and Health Sciences, Stellenbosch University, Cape Town, South Africa; 2 Division of Radiodiagnosis, Department of Medical Imaging and Clinical Oncology, Faculty of Medicine and Health Sciences, Tygerberg Hospital, Stellenbosch University, Cape Town, South Africa; 3 South African Health Informatics Association (SAHIA), Cape Town, South Africa; 4 Private Healthcare Information Standards Committee (PHISC), Cape Town, South Africa

**Keywords:** RIS, radiology information system, trauma, gun-shot, computerised tomography, crime, violence, Cape Town

## Abstract

**Background:**

South Africa (SA) has no national injury surveillance system, and hence, non-fatal gunshot injuries are not routinely recorded. Most firearm-related injuries require multi-detector computer tomography (MDCT) assessment at a tertiary-level facility. MDCT scanning for victims with gunshot injuries thus provide an indication of the societal burden of firearm trauma. The potential of the modern radiology information system (RIS) to serve as a robust research tool in such settings is not fully appreciated.

**Objective:**

The aim of this study was to evaluate the use of institutional RIS data in defining MDCT scanning trends for gunshot victims presenting to a tertiary-level SA hospital.

**Method:**

A single-institution, retrospective, comparative study was conducted at the Tygerberg Hospital (TBH) Trauma Unit for the years 2013 and 2018. Using data-mining software, customised RIS searches for information on all gunshot-related emergency computed tomography scans in the respective years were performed. Demographic, temporal, anatomical and scan-protocol trends were analysed by cross tabulation, Chi-squared and Fisher’s exact tests.

**Results:**

Gunshot-related emergency MDCT scans increased by 62% (546 vs. 887) from 2013 to 2018. Lower-limb CT angiography was the commonest investigation in both periods. A higher proportion of victims in 2018 sustained thoracic injuries (12.5% vs. 19.8%; *p* < 0.01) and required imaging of more than two body parts (13.1% vs. 19.2%; *p* < 0.01).

**Conclusion:**

By using RIS data to demonstrate the increasing gunshot-related MDCT workload in the review period, as well as a pattern of more complex and potentially life-threatening injury, this study highlights the burden of firearm trauma in the society and the potential role of the modern RIS as a robust research tool.

## Introduction

The deaths resulting from interpersonal violence have declined steadily in the new millennium from 144 000 in the year 2000 to 21 000 in 2018–2019 in South Africa (SA).^1,2^ This is arguably in response to the Firearms Control Act (FCA) of 2000, which was implemented in July 2004^3^ and introduced more stringent eligibility and competency requirements for firearm owners.^4^

Nonetheless, SA maintains the sixth highest homicide rate in the world (35/10^4^ people) and the second highest rate in sub-Saharan Africa.^5^ Furthermore, the Western Cape Province (WCP) is particularly violent, with 59 murders/10^4^ people and an apparent increase in the proportion of gun-related homicides. Cape Town was recently rated the eighth most dangerous city in the world.^4,6,7^

Allard and Burch^8^ revealed that 127 000 serious firearm-related injuries required emergency surgery at South African state hospitals during 2005. However, there has been no comparable national study in more than a decade. Of note, there has been no national injury surveillance system in SA. Non-fatal gunshot trauma is thus not routinely recorded.^8^

An overwhelming majority of firearm-related injuries require initial assessment at a tertiary-level healthcare facility. Modern multi-detector computed tomography (MDCT) scanning is the imaging modality of choice in this clinical setting. Scan protocols can be customised to accommodate any pattern of injury, thereby reflecting the extent of trauma.^9,10,11^ It was hypothesised that an analysis of computed tomography scans performed for gunshot-related injuries would provide a proxy assessment of the incidence and pattern of firearm trauma within a population. To the best of our knowledge, there has been no such study in any setting.

Developments in diagnostic imaging technology over the last half century have been paralleled by those in medical informatics. The perceived value of radiology as a medical specialty now extends well beyond the realm of pure diagnostics and includes digital information management.^12,13,14,15,16^ Numerous studies have documented the clinical benefits of the integrated picture archiving and communication system/radiology information system (PACS/RIS) in healthcare delivery, most notably the substantially higher productivity and efficiency, with lower consumable costs.^10,17,18,19^

The potential of the integrated RIS to serve as a robust research tool, through its capacity to store and manipulate complex image-related patient data, however, has received relatively limited attention. There is increasing recognition that RIS data have not been fully utilised. Initiatives in the last decade have refined data-mining techniques, making such data more readily accessible.^12^

Tygerberg Hospital (TBH) is a 1386-bed tertiary-level referral centre for approximately half of the population of the WCP. It is the main teaching hospital of the Faculty of Medicine and Health Sciences of Stellenbosch University in Cape Town, South Africa. It has a level-1 equivalent Trauma Unit that serves communities, with the highest reported incidence of gunshot murders in the WCP.^20,21^ It has a fully digital, filmless and paperless imaging department, with an integrated PACS/RIS, which was commissioned in 2012, and includes an electronic physician referral portal and embedded data-mining software.

The aim of this study was to evaluate the use of institutional RIS data in defining MDCT scanning trends for gunshot victims presenting to a tertiary-level SA hospital.

## Methods

A retrospective comparative study of emergency gunshot-related CT scans was performed in the TBH Trauma Unit for the calendar years 2013 and 2018. The embedded ‘Insite’ data-mining software was used to conduct customised searches of the TBH RIS (XIRIS 8.3 Phillips Medical Systems, The Netherlands) for the respective years. Details of CT scans with the terms ‘GSW’, ‘gun’, ‘bullet’, ‘firearm’ or ‘gunshot’ in the clinical history window of the electronic referral form were retrieved. CT scans not related to firearm-related injuries and follow-up scans performed after initial management of gunshot trauma were excluded from analysis. Data were anonymised, and stratified by patient demographics (age and gender), scan date (time, day and month) imaging protocol and anatomical region. The imaging protocol was defined by any combination of *body part* (brain, face, cervical spine, neck, upper limb, chest, thoracic spine, abdomen, pelvis, lumbar spine and lower limb) and additional *descriptor* (with contrast, CT angiography, IVP with/without cystogram and bony algorithm). Protocol analysis defined trends in the overall gunshot-related CT workload. The broader stratification of anatomical regions (head or neck, chest, abdomen or pelvis, upper limb and lower limb) was invoked to assess trends in the pattern of firearm-related injury and represents a modification of that reported by Norberg for forensic analysis of gunshot wounds.^22^ RIS data were also used to compare the number of firearm-related emergency CTs with the total number of scans performed in the TBH Trauma Unit and the overall CT workload of the TBH Radiology Department in 2013 and 2018. Institutional imaging protocols, CT scanning equipment and CT referral patterns were unchanged in the review period.

Summary statistics were reported as frequencies and percentages. Comparisons were made using cross tabulation, Chi-squared and Fisher’s exact tests. Statistica 13.5 was used for statistical analyses.

### Ethical consideration

The study was approved by the Health Research Ethics Committee of the Faculty of Medicine and Health Sciences, Stellenbosch University (Ref: S18/10/213). Patient anonymity was assured using unique study identifiers known only to the principal investigator. As this was a record-based retrospective study, patient management was not impacted in any way.

## Results

The TBH Trauma Unit performed 6700 MDCT scans in 2013, which increased to 7791 in 2018, representing a 16% overall and 2.7% average annual increase in caseload. Data mining for gunshot-related MDCTs yielded 704 and 1233 cases for 2013 and 2018, respectively. Approximately one-fifth of 2013 cases (158/704, 22.4%) and just over a quarter of 2018 cases (346/1233, 28.0%) were excluded, being follow-up scans, or examinations performed on patients with old gunshot injuries. Thus, emergency gunshot-related MDCT scans increased from 546 (average 46/month, 1.5 daily) in 2013 to 887 (average 74/month, 2.4 daily) in 2018, constituting a 62% overall and 10.3% average annual increase. By 2018, emergency gunshot victims represented 11% (887/7791) of the TBH Trauma Unit MDCT workload (Table 1).

**TABLE 1:** Tygerberg Hospital computed tomography scans.

TBH CT scans	Overall		Increase (%)	Female		Increase (%)	Male		Increase (%)
	2013	2018		2013	2018		2013	2018	
Total scans – all indications (*n*)	21 043	23 232	10	8480	9241	9	12 563	13 991	11
Trauma unit scans (*n*)	6700	7791	16	1443	1455	0.8	5257	6336	20.5
GSW scans: Trauma unit scans (*n*)	546	887	62	43	72	67	503	815	62
GSW scans: Trauma unit scans (%)	8	11	62	3	5	67	10	13	62
Average GSW scans/month	45.5	74	62	3.5	6.1	67	42	68	62
Average GSW scans/day	1.5	2.4	62	0.12	0.19	67	1.4	2.2	62
Total body parts scanned in GSW victims (*n*)	899	1577	75	62	119	92	837	1458	75
Average body parts scanned/GSW victim (*n*)	1.65	1.78	8	1.44	1.65	15	1.66	1.79	8
Number of different scan protocols (*n*)	124	155	25	20	28	40	122	144	18

CT, computed tomography; GSW, gun shot wound; TBH, Tygerberg Hospital.

Computed tomography angiography accounted for 46% (414/899) and 45% (717/1584) of total emergency gunshot-related workload in 2013 and 2018, respectively. Lower limb angiography was the commonest investigation performed in both periods, accounting for a quarter of body part scans in 2013 (222/899; 25%) and approximately one-fifth in 2018 (295/1584; 19%; Table 2).

**TABLE 2:** Gun shot computed tomography scan protocols.

GSW CT scans by protocol	Increase (%)
Brain	124
Face	46
C-spine	616
CTA neck	58
Chest	108
CTA chest	178
Urinary tract (IVP)	67
Abdomen	92
Pelvis	87
CTA pelvis	52
Cystogram	108
CTA upper limb	162
CTA lower limb	27

**Total**	**75**

CT, computed tomography; GSW, gun shot wound; CTA, computed tomography angiogram.

In addition to the overall increase in emergency gunshot victims, there were temporal scanning trends. In 2013, there was a wide monthly variation in scan volumes, with the highest monthly workload (*n* = 76) more than five times the lowest (*n* = 14). In 2018, caseload was more consistent, with scans in the busiest month (*n* = 97) exceeding those in the quietest month (*n* = 58) by a factor of < 2 (*p* = 0.00007; Table 3).

**TABLE 3:** Computed tomography scans by gunshot victims.

CTs for gunshot victims	Overall 2013				*p*	Female				*p*	Male				*p*
	2013		2018			2013		2018			2013		2018		
	*n*	%	*n*	%	*n*	%	*n*	%	*n*	%	*n*	%			
**Scan by gender**	546	-	887	-	n/a	42	7.9	72	8.1	0.91	503	92.1	815	91.8	0.91
**Scans by age**															
Age (IQR)	25	21–31	27	22–33	0.11	26	19–40	25	21–32	0.11	25	21–32	27	22–33	0.11
**Scans by month**	-	-	-	-	0.00007	-	-	-	-	0.06	-	-	-	-	0.00033
January	14	3	64	7	-	0	0	7	10	-	14	3	57	7	-
February	43	8	64	7	-	3	7	5	7	-	40	8	59	7	-
March	55	10	88	10	-	2	5	7	10	-	53	10.5	81	10	-
April	27	5	71	8	-	2	5	1	1.3	-	25	5	70	8.5	-
May	44	8	75	8	-	2	5	9	12.5	-	42	8	66	8	-
June	33	6	71	8	-	3	7	8	11	-	30	6	63	8	-
July	53	10	97	11	-	3	7	7	10	-	50	10	90	11	-
August	45	8	81	9	-	2	5	1	1.3	-	43	8	80	10	-
September	63	12	77	9	-	6	14	11	15	-	57	11	66	8	-
October	45	8	68	8	-	4	9	0	0	-	41	8	68	8	-
November	48	9	58	7	-	8	18	7	10	-	40	8	51	6.2	-
December	76	14	73	8	-	8	18	9	12.5	-	68	13.5	64	8	-
**Scans by day of week**															
Monday	104	19	118	13	0.16955	6	14	15	21	0.86	98	19	103	12.6	0.06988
Tuesday	69	13	110	12	-	5	12	3	4	-	64	13	107	13	-
Wednesday	55	10	97	11	-	7	16	8	11	-	48	9.5	89	11	-
Thursday	54	10	92	10	-	3	7	6	8	-	51	10	86	11	-
Friday	56	10	92	10	-	5	12	9	12.5	-	51	10	83	10	-
Saturday	96	18	175	20	-	8	18.6	15	21	-	88	17	160	19.6	-
Sunday	112	21	203	23	-	9	21	16	22	-	103	20.4	187	23	-
**Scans by time**															
Normal hours total	118	21	186	21	0.79	14	32.5	13	19	0.11	104	21	173	21	0.83
After hours total	428		701	-	-	29	67.5	58	81	-	399	79	648	79	-
**Scans by number of body parts**	-	-	-	-	< 0.01	-	-	-	-	0.05	-	-	-	-	0.05
One	296	54	447	51	-	25	58	43	42	-	271	54	404	51	-
Two	181	33	268	30	-	17	40	15	21	-	164	33	253	25	-
Three	43	8	116	13	-	1	2	10	14	-	42	8	109	13	-
Four	20	4	38	4	-	0	-	1	1	-	20	4	33	4	-
Five	4	1	16	2	-	0	-	2	3	-	4	0.8	13	1.5	-
Six	2	0.2	1	0.2	-	0	-	0	-	-	2	0.3	2	0.002	-
> 6	0	-	1	0.1	-	0	-	0	-	-	0		1	0.001	-
**Scans by anatomical region**															
Head and neck	118	21.6	203	22.6	0.52	8	18.6	19	26.4	0.37	110	21.8	184	22.6	0.73
Thorax	68	12.5	177	19.8	< 0.01	3	7.0	11	15.3	0.25	65	12.9	166	20.4	< 0.01
Abdo/pelvis	206	37.7	353	39.8	0.47	13	30.2	24	33.3	0.84	193	38.3	329	40.4	0.49
Upper limb	46	8.4	91	10.3	0.27	5	11.6	6	8.3	0.74	41	8.1	85	10	0.18
Lower limb	209	38.3	292	32.9	0.04	15	34.8	27	37.5	0.84	194	38.5	265	32.5	0.03

CT, computed tomography; IQR, interquartile range.

There were also trends in the anatomical pattern of injury. The key findings of this study were that a higher proportion of 2018 victims sustained thoracic trauma (12.5% vs. 19.8%; *p* < 0.01) and that a higher percentage required imaging of more than two body parts (13.1 vs. 19.2; *p* < 0.01), whilst a smaller proportion underwent lower limb scans (38.3 vs. 32.9; *p* = 0.04; Table 3).

Demographic features showed minimal variation, with males constituting just over 90% of victims in both 2013 and 2018 (*p* = 0.91), and the median age of the cohort being 25 and 27 years (*p* = 0.11) respectively. The weekly distribution of workload (*p* = 0.16955) and the proportion of cases presenting during normal working hours showed no significant variation (*p* = 0.79.)

Allowing for the small cohort of female victims, there was no significant gender-based differentiation in the pattern of injury (Table 3).

## Discussion

The embedded data-mining tool of an institutional RIS used to define trends in CT scanning for firearm-related injuries, highlighted the power of the RIS as a research tool, whilst contributing to the discourse on gun-related violence in the society.

Through a simple series of RIS searches and subsequent analyses, compelling evidence was provided of the relentless increase in firearm violence in certain Cape Town suburbs. The findings also suggest evolution in the annual pattern of gunshot injuries from cyclical to sustained throughout the year. Additionally, the demonstration of a significant increase in the proportion of victims undergoing scans of more than two body parts suggests an escalation in multiple gunshot wounds. The significant increase in chest scans, with a corresponding decrease in those of the lower limbs, suggests an evolving pattern of more life-threatening injury. The particular benefit and context of this work is that SA has no national injury surveillance system, with non-fatal gunshot trauma not routinely or comprehensively recorded. Additionally, there has been no comparable RIS-based study of epidemiological trends in any setting.

This study interfaces with the domains of public health, emergency medicine, traumatology, health economics and health informatics, demonstrating the role of the RIS in transversal interdisciplinary research. Of note, despite their great abundance, RIS data are not readily available or immediately useful. Like all corporate databases, the RIS is ‘data rich but information poor’, with information typically archived in unstructured format. The free text of the diagnostic report is a prime example. A mechanism for data extraction and conversion to a useful format is thus essential. In this study, a keyword search was conducted of unstructured referral details. Retrieved data were then exported as a Microsoft Excel spreadsheet, facilitating subsequent analysis.

The limitations inherent in unstructured RIS data can be mitigated by generating coded data. The International Statistical Classification of Diseases and Related Health Problems (ICD) stratification of the World Health Organization (WHO) is extensively used to assist in data retrieval from a range of databases. Although the latest iteration, the ICD-10 codes, is widely used in SA for medical billing, their potential role in academic radiology reporting and research has not been explored. Standardised, structured and coded RIS data would make a substantial contribution to radiology research in academic departments, nationally.

This research article is particularly relevant, considering the increasing global conversion to digital radiological services. There is ongoing commissioning of integrated PACS/RIS platforms, particularly in low- and middle-income countries. This is certainly true in the public healthcare sector in SA, most notably in academic radiology departments. When undertaking analogue to digital conversion, there is a tendency to focus on the PACS component, with scant attention being paid to the RIS, thereby risking acquisition of a RIS without data-mining ability. Such capability is not standard in entry-level solutions and typically requires clear specification at the time of procurement. Failure to secure such functionality at the outset can be costly, either financially, if one purchases an add-on solution, or in a lost research opportunity. Additionally, add-on programs tend to be less effective than embedded, customised solutions.^23^

Data mining is one of the aspects of advanced RIS functionality. A pivotal additional ability is its integrative role with other information systems, particularly the PACS, the hospital information system and the electronic medical record. Further functions to be considered at the time of RIS procurement include clinical decision support, radiologist workflow management and departmental quality metrics.^24^

This study was limited by its retrospective design. However, the impact was offset by the widely acknowledged, robust and comprehensive nature of RIS databases, as well as the use of focused search terminology, which is closely aligned with the institutional referral vocabulary. The accuracy of such a strategy for analysing free text, stand-alone radiological reports has been documented.^25^ A further limitation was that the extent of gunshot-related organ injury was not included in the research question. However, this study has afforded full appreciation of the capacity of the RIS to serve as a platform for further studies to evaluate trends in the severity of gunshot injuries as defined by baseline imaging findings.

## Conclusion

Using RIS data to demonstrate the increasing emergency gunshot-related MDCT workload in the review period, as well as a pattern of more complex and potentially life-threatening injury, this study highlights the burden of firearm trauma on society and the potential role of the modern RIS as a robust research tool.
